# Early handwriting development: a longitudinal perspective on handwriting time, legibility, and spelling

**DOI:** 10.3389/fpsyg.2024.1466061

**Published:** 2025-01-08

**Authors:** Lidia Truxius, Judith Sägesser Wyss, Michelle N. Maurer

**Affiliations:** ^1^Institute of Psychology, University of Bern, Bern, Switzerland; ^2^Institute of Special Needs Education, Bern University of Teacher Education, Bern, Switzerland; ^3^Norwegian Reading Centre, University of Stavanger, Stavanger, Norway

**Keywords:** handwriting time, handwriting legibility, spelling, children, development

## Abstract

**Introduction:**

Learning to write is a complex task involving peripheral (e.g., handwriting speed and legibility) and central (e.g., spelling) processes. Coordinating these processes is particularly demanding for novice writers who have not yet automated their handwriting skills. To better support children in developing handwriting, it is crucial to understand the development and interactions of these peripheral and central processes over time.

**Methods:**

This longitudinal study (*n* = 363; 49.8% girls) investigated the development and interrelations of handwriting speed (time spent on writing tasks), legibility, and spelling in German-speaking first-grade children (*M*_age_ = 7 years) across 12 months. The children were assessed at three time points, spaced 6 months apart, from the beginning of the first grade to the start of the second grade.

**Results and discussion:**

While performance in all domains of handwriting (time, legibility, and spelling) improved over the school year, these skills were particularly strongly interrelated at the beginning of writing acquisition but became increasingly independent towards the second grade. Surprisingly, the results from the structural equation model showed that the relations between handwriting legibility and time reversed over time: Initially, faster handwriting was associated with more legible handwriting, while with increasing practice a trade-off appeared. Furthermore, when considering cross-lagged paths, the structural equation model revealed that handwriting legibility at the beginning of the first grade significantly predicted subsequent handwriting time and spelling abilities at the end of the school year. In summary, handwriting proficiency stabilizes quickly, while patterns of associations between peripheral and central handwriting processes change across the first year of handwriting instruction.

## Introduction

Writing is an essential tool for conveying thoughts and preserving knowledge, involving a sophisticated interplay of cognitive and motor processes. Composing written content requires individuals to convert abstract ideas into words that adhere to spelling rules. At the same time, fine motor control of finger and hand movements is essential for producing legible text. More recently, two facets of writing have been discussed: the central processes, which involve retrieving and manipulating orthographic knowledge for spelling, and the peripheral processes, which cover the motor aspects of letter formation (Afonso and Álvarez, 2019). For novices, such as young children learning to write, both domains are new. Early on, they must simultaneously coordinate the processes of forming legible characters efficiently (peripheral processes) and following phoneme-grapheme rules (central processes; Afonso and Álvarez, 2019). Although children dedicate considerable portions of their school day to writing tasks, there is a scarcity of research focused on how fundamental writing skills, such as spelling, handwriting speed (i.e., time spent on handwriting tasks), and legibility, emerge and interrelate in beginner writers but see Caramia et al. (2020). Understanding these fundamental skills and their interactions is imperative for fostering literacy and education.

Theoretical writing models, such as the Not-so-Simple View of Writing (Berninger and Winn, 2006), posit that transcription skills, such as handwriting and spelling abilities, must become automatic to liberate cognitive resources for higher-order writing tasks like text generation. While this model is widely accepted and supported by diverse research (Berninger et al., 2010; Salas and Silvente, 2020; Valcan et al., 2020), it cannot explain the interrelations between the individual transcription skills (i.e., handwriting and spelling) and how these develop to reach automaticity.

Alternatively, van Galen’s (1991) psychomotor model views writing as a multicomponent task that integrates cognitive, psychomotor, and biophysical processes. Cognitive processes manage idea generation and word retrieval, tapping into long-term memory for spelling rules and letter shapes. Meanwhile, psychomotor processes regulate pen movements, complemented by biophysical mechanisms that adjust pressure and timing, thus ensuring consistency in the written output (e.g., letter shapes). In a hierarchical framework, the result of a higher-level process initiates subsequent, lower-level processes. For instance, novice writers must recall the correct letter sequence from memory (higher order) and convert this into precise motor movements to form this letter sequence on the paper (lower order).

Many studies estimate handwriting proficiency using the alphabet writing task, which measures the speed of recalling and handwriting the letters of the alphabet (e.g., Kim et al., 2013; Valcan et al., 2020). This task necessitates remembering the letters in their correct order and is influenced, among other factors, by a child’s knowledge of the alphabet and the speed at which they can retrieve this information from memory. However, measures of handwriting speed that account for time spent writing in a more naturalistic context are rare yet necessary to better understand the development of handwriting speed (i.e., time), legibility, and spelling, as well as how these aspects interact over time.

Moreover, there is a scarcity of studies investigating handwriting legibility and handwriting time simultaneously (however, see: Gosse et al., 2021; Graham et al., 2011; Karlsdottir and Stefansson, 2002; Rosenblum et al., 2003). Legible handwriting is crucial for readers’ decoding and reading comprehension and affects how teachers view assignments. Neater and more legible handwriting often leads to higher grades, independent of the essay’s content (Graham et al., 2011; Rosenblum et al., 2003). Furthermore, efficient handwriting, that is, quick handwriting (Salas and Silvente, 2020; Valcan et al., 2020), is vital for staying on track with classroom assignments and activities, as in many contexts, more efficient handwriting results in a longer final product (Malpique et al., 2020; Suggate et al., 2018).

Differing from skilled writers, children at the beginning of handwriting acquisition who have not yet automated the skill (Fitjar et al., 2021) must switch their attention between various writing processes (Olive and Kellogg, 2002). Studies in elementary school students show that the fundamental central (e.g., spelling) and peripheral writing processes (e.g., time and legibility) might interact, as the requirements of one skill (e.g., spelling) can influence the other (e.g., time). For example, 8-10-year-old children displayed longer handwriting times when dealing with orthographically irregular words as opposed to orthographically regular ones, suggesting that increased spelling demands require children to reduce their handwriting speed (Kandel and Perret, 2015). Additional evidence of a link between peripheral and central writing processes has been found in research on bilingual children, who exhibit less legible handwriting than their monolingual peers, pointing to a potential connection between spelling complexities and legibility (Caravolas et al., 2020).

The relations between handwriting speed and legibility, however, present varied evidence: Certain studies indicate a positive correlation, with more legible handwriting aligning with faster handwriting, and conversely, less legible handwriting going along with slower handwriting (Fitjar et al., 2022; Maurer, 2023). In contrast, other studies point to a negative correlation, with more legible handwriting being associated with more time spent on handwriting, implying that children slow down their handwriting to enhance precision (Gosse et al., 2021). Some studies even suggest that speed and legibility are distinct, unrelated elements (Duiser et al., 2020; Downing and Caravolas, 2023). These mixed findings point to our fragmented understanding of the development of this trade-off between legibility and handwriting time in novice writers.

To get a comprehensive picture of how central and peripheral processes develop and interact, handwriting time, legibility, and spelling need to be investigated simultaneously. Few studies so far have done this. A recent concurrent study explored these domains in third-, fourth-, and fifth-grade children, concluding that graphomotor skills are more crucial for handwriting legibility, while spelling plays a more significant role in handwriting speed (Downing and Caravolas, 2023). Gosse et al. (2021) confirmed a link between spelling and handwriting speed and found that early spelling proficiency in third grade predicted subsequent handwriting speed, indicating a steeper increase in handwriting speed in children with initially poorer spelling skills. In third grade, a significant negative association was observed between handwriting legibility and time, suggesting that more legible handwriting goes along with slower handwriting. However, no longitudinal relations were identified between handwriting time, legibility, and spelling, possibly due to the well-developed handwriting skills typical in late elementary school. Although speed and spelling tend to improve throughout elementary school (Karlsdottir and Stefansson, 2002; Gosse et al., 2021), handwriting legibility seems to plateau after second grade (Karlsdottir and Stefansson, 2002; Overvelde and Hulstijn, 2011). Research on younger children aged 5–6 years suggests that legibility might support spelling development (Pritchard et al., 2021), yet the long-term dynamic between these writing elements (i.e., time, legibility, and spelling) in novice writers remains elusive.

### The present study

This study aims to investigate the development and longitudinal relations between handwriting time, legibility, and spelling in young, novice writers. We assessed children’s handwriting time and legibility three times across the first year of handwriting instruction. Additionally, spelling abilities were assessed twice, at the end of first grade and the beginning of second grade. Based on van Galen’s (1991) psychomotor writing model and prior findings, we anticipate concurrent and longitudinal interrelationships between these writing skills, hypothesizing that more efficient (i.e., less time spent on a handwriting task and thus faster) handwriting correlates with higher spelling accuracy and vice versa. However, uncertainties persist about how handwriting time and legibility interrelate in novice writers (Gosse et al., 2021; Downing and Caravolas, 2023; Maurer, 2023), so we did not formulate any hypothesis.

## Methods

### Participants

A sample of 363 children (49.8% girls) from diverse urban and rural areas in Switzerland underwent three assessments: at the start of the first grade (T1; *M* = 7 years, *SD* = 4.65 months, range = 6 years 3 months to 8 years 2 months), the end of first grade (T2), and at the beginning of the second grade (T3), each assessment being 6 months apart. The majority (86%) were right-handed, and 40% received varied educational support, such as speech and language therapy, psychomotor therapy, special language support for non-native speakers, and occupational therapy, which is common for children needing assistance in meeting learning objectives in Switzerland. Most of the children (75%) spoke German/Swiss-German. All children were taught writing in German. German is a language with low orthographic depth, allowing children to spell the words letter by letter (Seymour et al., 2003). Socioeconomic background was assessed using the International Socio-Economic Index (ISEI; Ganzeboom et al., 1992) based on parental occupations, considering income and education, and varied widely from 14.39 to 88.98 (low-status jobs like waste disposal to high-status professions such as judges). Mothers’ average socioeconomic index was *M* = 54.60 (*SD* = 20.05), and fathers’ *M* = 56.88 (*SD* = 21.63), both slightly above the European average (Entorf and Minoiu, 2005). Written consent was obtained from the parents, and verbal consent from the children. This study, approved by the Ethics Committee of the Bern University of Teacher Education, was part of a broader research project (Approval No. 19s000201).

### Measures

#### Handwriting time

To assess handwriting time, children copied six words (four six-letter words and two eight-letter words) on a piece of paper, placed on a digitized tablet using a WACOM Inking Pen. Light grey bars of 1 cm in height were printed on the paper to ensure that children wrote in a comparable size. The digitized tablet, connected to a laptop computer, utilized the software CSWin (Mai and Marquardt, 2016) to capture the duration of writing (i.e., handwriting time). Specifically, time in milliseconds was recorded from the first pen contact at the start to its lift-off after completion.

#### Handwriting legibility

The same six words used for handwriting time assessment were rated for overall legibility (Barnett et al., 2018). A co-author independently rated the recognizability of each letter within a word, determining if it could be identified as this letter in isolation (score 1) or if it could not be identified or could be mistaken for another letter (score 0)—without the context provided by adjacent letters. This global, i.e., holistic evaluation is a standard method in many handwriting legibility studies (see Rosenblum et al., 2003 for review). To accommodate words of varying letter counts, legibility was calculated as the percentage of legible letters per word.

#### Spelling

Children’s spelling abilities were evaluated using four words of varying difficulty from the Hamburger Writing Test [Hamburger Schreibprobe] (May, 2002), a standardized test for first and second graders. The words were presented orally with accompanying images. Children spelled the words on paper, and accuracy was scored as the percentage of correctly written graphemes for each word. Spelling was assessed at the second (T2; end of first grade) and third (T3; start of second grade) time points but not initially (T1) since children at the start of first grade generally cannot spell.

### Data analysis

Initially, a repeated measures ANOVA for the three writing variables across the different assessment points was performed using RStudio Version 4.3.3 and the package Jamovi (The Jamovi Project, 2024). Subsequently, we conducted correlation analyses for the three writing variables across the different assessment points. The analyses utilized the mean handwriting time and the percentage of correct letters (i.e., graphemes) across all words. In the repeated measures ANOVA, generalized Eta squared was employed to estimate effect sizes, with η^2^ = 0.01 indicating a small effect, η^2^ = 0.06 a medium effect, and η^2^ = 0.14 a large effect (Bakeman, 2005; Cohen, 1973).

To investigate the longitudinal relationships among handwriting time, legibility, and spelling abilities, we conducted a cross-lagged structural equation model using MPlus Version 8.7 (Muthén and Muthén, 2017). Full maximum likelihood estimation was used to handle missing data, and we allowed covariances to vary to accommodate shared variances between measures. Criteria for good model fit are defined by an insignificant Chi-square (χ^2^), CFI > 0.95, RMSEA < 0.08, and SRMR < 0.06 (Hu and Bentler, 1999). As Chi-square is sensitive to sample size (Cheung and Rensvold, 2002; Curran et al., 2002), in larger samples, an appropriate Chi-square can also be defined by χ^2^/df < 2 (Jöreskog, 1993). Mothers’ and fathers’ socioeconomic backgrounds (measured by ISEI) were not significantly related to any of the writing variables (correlations ranging between *r* = 0.00 and *r* = 0.11) and were therefore not further considered in the analyses.

## Results

### Mean differences in handwriting development

Table 1 shows the descriptive statistics for the handwriting variables across the three measurement points, as well as mean difference tests across the measurement points, as indicated by the results of the repeated measures ANOVA. Performance on all handwriting variables changed significantly across the first and second school years. Post-hoc pairwise comparisons using the Tukey test for handwriting time and handwriting legibility revealed significant changes between the first and second [time: *t*(312) = 23.78, *p* < 0.001 legibility: *t*(307) = 5.39, *p* < 0.001], the second and the third [time: *t*(312) = 15.63, *p* < 0.001; legibility: *t*(307) = 5.30, *p* < 0.001], and the first and the third measurement points [time: *t*(312) = 33.86, *p* < 0.001; legibility: *t*(307) = 9.36, *p* < 0.001]. Table 2 presents correlations between the handwriting variables over the three measurement points.

**Table 1:** Means (M), standard deviations (SD), and repeated measures ANOVA.

	Measurement					
	T1	T2	T3			
	*M* (*SD*)	*M* (*SD*)	*M* (*SD*)	*F* (df1, df2)	*p*	Generalized η^2^
Handwriting time (in ms)	29,246.49 (9,161.28)	17,697.98 (6,367.987)	13,698.66 (4,899.68)	757.90 (2, 312)	<0.001	0.46
Handwriting legibility (% correct)	85.96% (10.27%)	89.00% (8.44%)	91.58% (7.96%)	51.78 (2, 307)	<0.001	0.06
Spelling (% correct)	-	79.34% (11.56%)	82.91% (9.98%)	67.20 (1, 331)	<0.001	0.03

**Table 2:** Pearson correlations for the manifest writing variables.

	1	2	3	4	5	6	7
1. Handwriting time T1	-	-	-	-	-	-	-
2. Handwriting time T2	0.47***	-	-	-	-	-	-
3. Handwriting time T3	0.49***	0.66***	-	-	-	-	-
4. Handwriting legibility T1	−0.16**	−0.20***	−0.15**	-	-	-	-
5. Handwriting legibility T2	−0.15**	−0.04	−0.07	0.41***	-	-	-
6. Handwriting legibility T3	−0.14*	−0.01	0.08	0.39***	0.52***	-	-
7. Spelling T2	−0.12*	−0.30***	−0.24***	0.34***	0.33***	0.22***	-
8. Spelling T3	−0.12*	−0.28***	−0.27***	0.29***	0.39***	0.22***	0.68***

*p < 0.05, **p < 0.01, ***p < 0.001.

### Longitudinal trajectories of handwriting development

Longitudinal trajectories of handwriting development were analyzed using a cross-lagged structural equation model, which encompassed handwriting time, legibility, and spelling abilities at three different measurement points. The model fits the data well: *χ*^2^(840) = 1244.70, *p* < 0.001, CFI = 0.94, RMSEA = 0.04, SRMR = 0.05. The Chi-square difference test was significant, with *χ*^2^/df = 1.48, which falls within acceptable benchmarks for a reasonable Chi-square value (Jöreskog, 1993). Factor loading variability for the manifest variables on latent constructs at each time point is shown in Table 3. Manifest variables consisted of scores from six written words for handwriting time and legibility and scores from four written words for spelling abilities. All factor loadings surpassed the accepted thresholds (Chin, 1998).

**Table 3:** Factor loadings of the manifest variables.

	Measurement point		
	T1	T2	T3
	λ	λ	λ
Handwriting time (six variables)	0.78–0.89	0.76–0.85	0.74–0.84
Handwriting legibility (six variables)	0.45–0.79	0.52–0.66	0.51–0.77
Spelling abilities (four variables)	-	0.49–0.70	0.40–0.58

**Figure 1:**
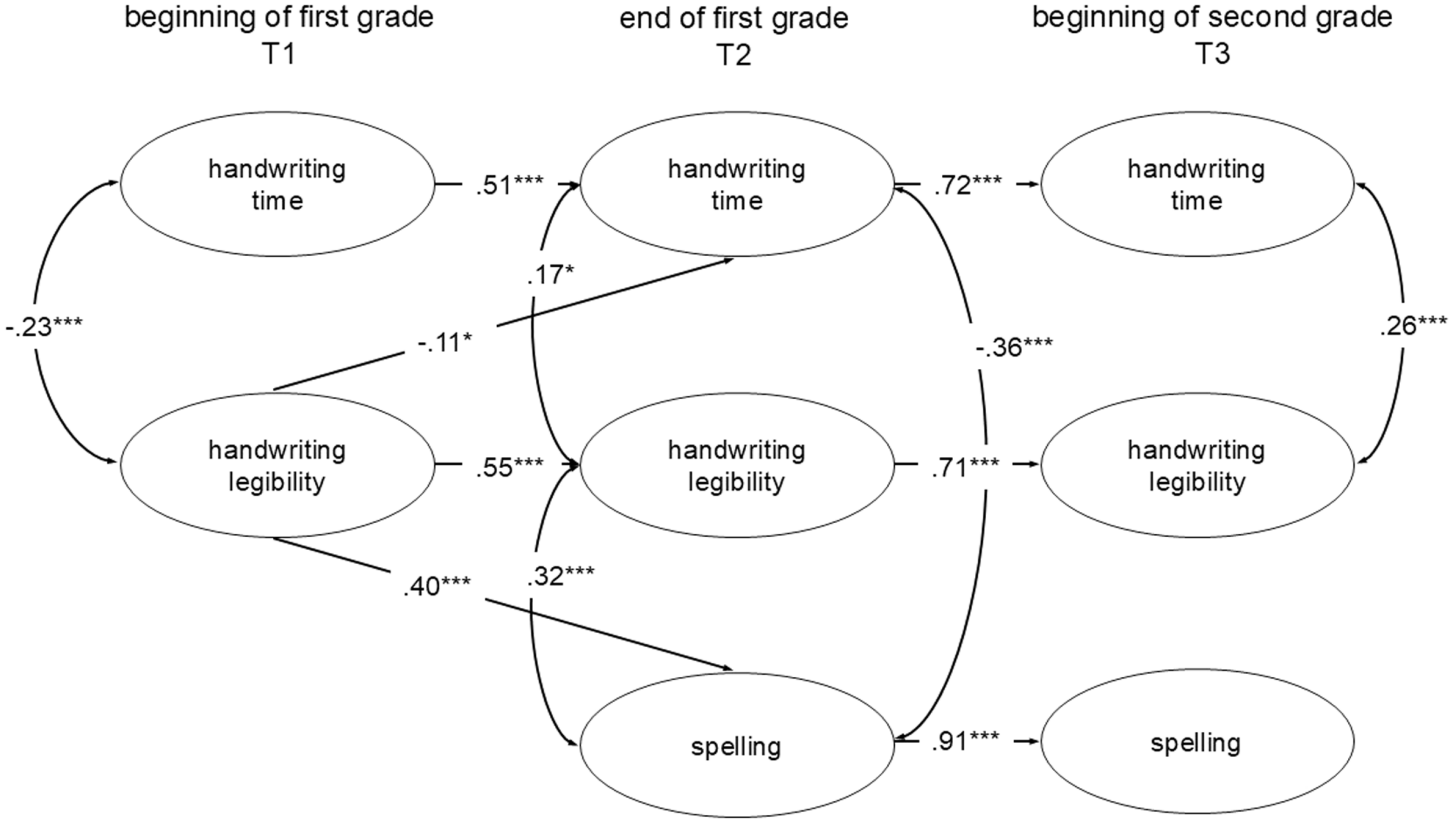
Structural equation model of the peripheral processes (i.e., time and legibility) and central processes (i.e., spelling) across three measurement points. The oval shapes represent latent variables. Only significant paths are shown for clarity (*p < 0.05, **p < 0.01, ***p < 0.001).

Figure 1 presents the standardized path coefficients for the estimated cross-lagged structural equation model. While all paths were estimated, including cross-sectional correlations, only significant paths are depicted in the figure. At the onset of first grade (T1), a negative association was found between handwriting time and legibility (*r* = −0.23, *p* < 0.001), suggesting that writers who needed less time tended to write more legibly. In contrast, by the end of first grade (T2; *r* = 0.17, *p* = 0.02) and the start of second grade (T3; *r* = 0.26, *p* = 0.001), the relationship reversed, and children who spent more time on the handwriting task wrote more legibly.

Furthermore, handwriting time correlated with spelling abilities at the end of first grade (T2; *r* = −0.36, *p* < 0.001), suggesting that children who spent less time on the handwriting task spelled more accurately, but this association was not evident at the beginning of second grade (T3; *r* = −0.28, *p* = 0.24). Additionally, legible handwriting was associated with better spelling abilities (T2; *r* = 0.32, *p* < 0.001).

From a longitudinal perspective, significant pathways were identified from legibility at the beginning of first grade to both handwriting time (β = 0.11, *p* = 0.04) and spelling abilities (β = 0.40, *p* < 0.001) at the end of first grade, indicating that more legible handwriting initially may contribute to later improvements, in both, time and spelling.

## Discussion

This study examined both concurrent and longitudinal relations among central (i.e., spelling) and peripheral (i.e., handwriting time and legibility) facets of writing in children during the first year of handwriting instruction. Findings indicated that handwriting time, legibility, and spelling were strongly associated at the start of first grade; as instruction continued, these skills improved and stabilized throughout the first year. Longitudinally, handwriting legibility emerged as a particularly important predictor of subsequent handwriting time and spelling abilities.

At the beginning of first grade, when writing instruction had just started, better legibility was associated with more efficient handwriting (e.g., less time spent on writing task). As children received more practice and instruction, this relationship reversed, and better legibility tended to coincide with longer handwriting times. Initially, children exhibited an overall competence in handwriting that included both legibility and time, which is in line with previous research on beginner writers (Fitjar et al., 2021). However, as instruction progressed, children appeared to place greater emphasis on the coordination of legibility and time, slowing down to improve letter formation accuracy. This balancing act or trade-off between accuracy and speed is commonly seen in more skilled writers, including older children and adults (Gosse et al., 2021; Graham, 2018). By the end of the first grade, children could modulate their writing speed based on the task demands, demonstrating an emerging level of writing proficiency. This finding can also be interpreted by van Galen’s psychomotor model. When children at the beginning of handwriting copy letters and strokes, higher-level processing skills are scarcely involved. However, with expanding letter and vocabulary knowledge, phonological and semantic abilities build an additional processing level, requiring more cognitive resources—which is reflected in a trade-off between legibility and speed.

It is important to note that we only considered handwriting time in terms of time spent on the writing task. However, other aspects of handwriting fluency are relevant to handwriting proficiency (for an overview see Truxius et al., 2024; Gargot et al., 2020). For instance, Fitjar et al. (2022) suggest that the number of velocity peaks could be a more significant indicator of handwriting legibility than time because varied acceleration and deceleration within a single stroke can reduce consistency in letter formation. As such, the relationship between handwriting fluency and legibility may differ from that between handwriting time and legibility. Software applications that can precisely measure indicators of handwriting fluency, such as velocity peaks, provide further insights into the fine motor processes underlying handwriting difficulties or development in handwriting speed and legibility (Paz-Villagrán et al., 2014; Truxius et al., 2024). Nevertheless, the time a child spends on a handwriting task continues to be a significant metric within the educational environment, where in most cases, more efficient handwriting is linked with longer texts and the ability to keep pace with the academic demands and assignments.

Moreover, handwriting legibility was associated with spelling abilities at the end of the first grade, but this association vanished at the beginning of the second grade. This observation is consistent with previous studies that report a relationship between handwriting legibility and spelling in the early stages of writing proficiency, which seems to decrease as children practice and the processes become more independent (Gosse et al., 2021; Pritchard et al., 2021). A similar trend was observed in the relation between handwriting time and spelling. Initially, in the first grade, better spelling abilities corresponded with shorter handwriting time; however, by the second grade, this link was no longer evident. There are at least two possible explanations for the increasing independence of spelling skills from handwriting: Firstly, as handwriting becomes more automated, cognitive resources are liberated for spelling, as postulated by the Not-so-Simple-View of Writing (Berninger and Winn, 2006), resulting in spelling and handwriting time becoming more independent of each other. Secondly, improved spelling abilities may allow children to recall letters from memory more quickly, leading to shorter handwriting time and better legibility. This notion is reinforced by evidence suggesting that phonological awareness and letter knowledge facilitate writing development (Puranik et al., 2011; Frolek Clark and Luze, 2014). Additionally, considering that children with initially lower spelling skills showed more significant improvements in handwriting over time (Gosse et al., 2021), it could be inferred that once children reach a certain level of proficiency in letter retrieval, spelling no longer influences handwriting time and legibility. Consequently, spelling and handwriting skills become independent of each other.

Although the processes become more independent with practice and the writing processes stabilize over time, early handwriting legibility — reflecting the ability to produce letters accurately, which requires fine motor control — predicts later handwriting time and spelling abilities. Since handwriting legibility is associated with fine motor control (Volman et al., 2006; Kaiser et al., 2009) and depends on the consistent formation of letters over time (Di Brina et al., 2008), it plays a critical role in the development of fluent and automatized handwriting. Practicing and internalizing fine motor movements most likely leads to shorter handwriting times. Moreover, as better fine motor skills, particularly graphomotor skills, are linked to better spelling abilities (Dinehart and Manfra, 2013; Maurer et al., 2023), it is not surprising that early handwriting legibility at the onset of handwriting instruction is beneficial to enhancing spelling abilities.

While the findings of this study offer valuable insights into the writing development of young children, it is essential to recognize certain limitations that may affect the interpretation and generalizability of the results. First, considering that children in this study wrote individual words (as opposed to sentences), our assessment was limited to global ratings of words. We did not account for other elements, such as spacing, punctuation, size, and alignment, which are further aspects of legibility. Future research should aim to measure handwriting within a more comprehensive context, like sentences or texts, for a more thorough assessment of handwriting legibility. Secondly, due to the study’s focus on specific central and peripheral writing processes, the role of other potentially relevant lower-level writing processes, such as phonological and semantic processing abilities, remains unknown. A third limitation concerns the assessment of spelling skills, which occurred at only two measurement points, as children could not yet spell words at the onset of the first grade. Consequently, since we could not evaluate spelling skills at the beginning of the first grade, it is likely that the longitudinal relationships might be weaker if prior spelling skills were taken into account.

This study reveals that handwriting time, legibility, and spelling are most closely related at the beginning of handwriting acquisition but become more independent with practice. Handwriting proficiency stabilizes quickly in the first year, though the relations between peripheral and central handwriting processes vary. Notably, there is a trade-off between handwriting time and legibility, which reverses over the first school year.

These findings highlight the complexity of early handwriting and the evolving associations between handwriting processes at the very beginning of learning to write. Future research should explore the dependencies between further writing processes, as suggested by van Galen (1991), to better understand their role in developing automated handwriting.

## Data Availability

The data and code necessary to reproduce the analyses presented here are publicly accessible at OSF at the following URL: https://osf.io/5tx6d/?view_only=c2f6de9bed7543e5a6ec6dfe7d532d5e.
